# Inverse Boltzmann Iterative Multi-Scale Molecular Dynamics Study between Carbon Nanotubes and Amino Acids

**DOI:** 10.3390/molecules27092785

**Published:** 2022-04-27

**Authors:** Wanying Huang, Xinwen Ou, Junyan Luo

**Affiliations:** 1 T-Life Research Center, State Key Laboratory of Surface Physics, Department of Physics, Fudan University, Shanghai 200433, China; huangwanying1128@163.com; 2 Department of Chemistry, The Hong Kong University of Science and Technology, Clear Water Bay, Kowloon, Hong Kong 999077, China; xwou@ust.hk; 3 Department of Physics, Zhejiang University of Science and Technology, Hangzhou 310023, China

**Keywords:** multi-scale molecular dynamics, IBI, CBNs

## Abstract

Our work uses Iterative Boltzmann Inversion (IBI) to study the coarse-grained interaction between 20 amino acids and the representative carbon nanotube CNT55L3. IBI is a multi-scale simulation method that has attracted the attention of many researchers in recent years. It can effectively modify the coarse-grained model derived from the Potential of Mean Force (PMF). IBI is based on the distribution result obtained by All-Atom molecular dynamics simulation; that is, the target distribution function and the PMF potential energy are extracted, and then, the initial potential energy extracted by the PMF is used to perform simulation iterations using IBI. Our research results have been through more than 100 iterations, and finally, the distribution obtained by coarse-grained molecular simulation (CGMD) can effectively overlap with the results of all-atom molecular dynamics simulation (AAMD). In addition, our work lays the foundation for the study of force fields for the simulation of the coarse-graining of super-large proteins and other important nanoparticles.

## 1. Introduction

In recent years, molecular dynamics simulation has become an important bridge for the combination of experimental research and theoretical research [1,2,3]. In particular, the use of molecular dynamics to study the molecular dynamics interaction between proteins and nanoparticles [4,5] has attracted close attention, including within the biopharmaceuticals field [6,7,8]. At present, in the process of developing new drugs, the use of molecular dynamics for early-stage drug targeted screening has become an important part of new drug development [9]. Even due to the rapid development of nanotechnology, some research related to nanotechnology security has received extensive attention [4,10,11,12,13]. However, with the continuous improvement of computer computing power, especially the simulation calculation with the assistance of high-performance graphics GPU, the system that MD can simulate is becoming bigger and the simulation time is becoming longer. Much work in molecular dynamics still only calculates the relationship between local globulins and associated ligands. The mechanism of interaction between nanoparticles and supersized proteins is still unclear. In recent years, coarse-grained molecular dynamics simulation, as a pioneer in exploring the simulation of super-large and complex systems, has been playing a unique role [3,14,15].

The development between coarse-grained simulation (CG) and all-atom simulation has always been complementary and spiral development [16,17,18]. Among them, the coarse-grained simulated force field represented by Martin (The MARTINI coarse-grained force field) provides a very effective coarse-grained structure for molecular dynamics transmembrane research. In recent years, the multi-scale coarse-grained model has been an important supplement to the traditional coarse-grained simulation, especially several multi-scale simulation methods based on molecular dynamics simulation to extract the force field, IBI, Energy Decomposition, Fluctuation Matching method, etc. They are derived from the conformational distribution and energy or force derived from the all-atom simulation. Among them, the IBI multi-scale simulation method, as a modification of the classical mean-field coarse-grained force field, is a very effective coarse-grained force field extraction scheme.

IBI is a multi-scale simulation method that has attracted the attention of many researchers in recent years [19,20,21,22,23]. It can effectively modify the coarse-grained model derived from the mean field. IBI is based on the distribution result $g_{Target}\left(r\right)$ obtained by All-Atom (AA) molecular dynamics simulation. The PMF potential energy is extracted [24,25], and it is then used as the initial potential energy for the iterative process of IBI. IBI initially had a very good performance in fluid coarse-grained simulation. Then, it plays a key role in the research and simulation system of polymers. At present, IBI has been in the field of super-large polymer system research [26,27,28], as represented by the German scholar Muller [29], and much excellent research work has emerged. In addition, some researchers use IBI to conduct research such as transmembrane research and coarse-grained simulation research of DNA and super-large proteins. The interaction between amino acids and nanoparticles has been receiving attention from many researchers [30,31,32,33,34]. It is the preliminary work of the adsorption of super-large proteins and nanoparticles [35]. Therefore, in our research work, it is very appropriate to choose IBI as the research method for the extraction of carbon nanotubes and 20 kinds of amino acid force fields. It should be noted that there are many excellent multi-scale simulation software, such as VOTCA [36]. VOTCA [36] is a pretty good multi-scale simulation software, but we did not use it for the following reasons: (a) our research system is special, which examines the interaction between rigid bodies and flexible systems; (b) we may introduce special light fields in the future; (c) it may be necessary to use machine learning to improve the fitting efficiency of IBI; and (d) research on the interaction between some special particles and proteins. Due to the above research requirements, we hope that using our own CGMD program will bring more possibilities for the development of research.

Our work investigated the coarse-grained interaction between 20 amino acids and the representative carbon nanotube CNT55L3. More than 100 simulation iterations were performed using IBI, and finally, the distribution obtained by the coarse-grained simulation (CGMD) can effectively overlap with the result of the all-atomic molecular dynamics simulation (AAMD). In addition, our work lays the foundation for force field research for the simulation of coarse-grained super-large proteins and other important nanoparticles.

## 2. Simulation System and Research Method

### 2.1. Simulation System

**Figure 1:**
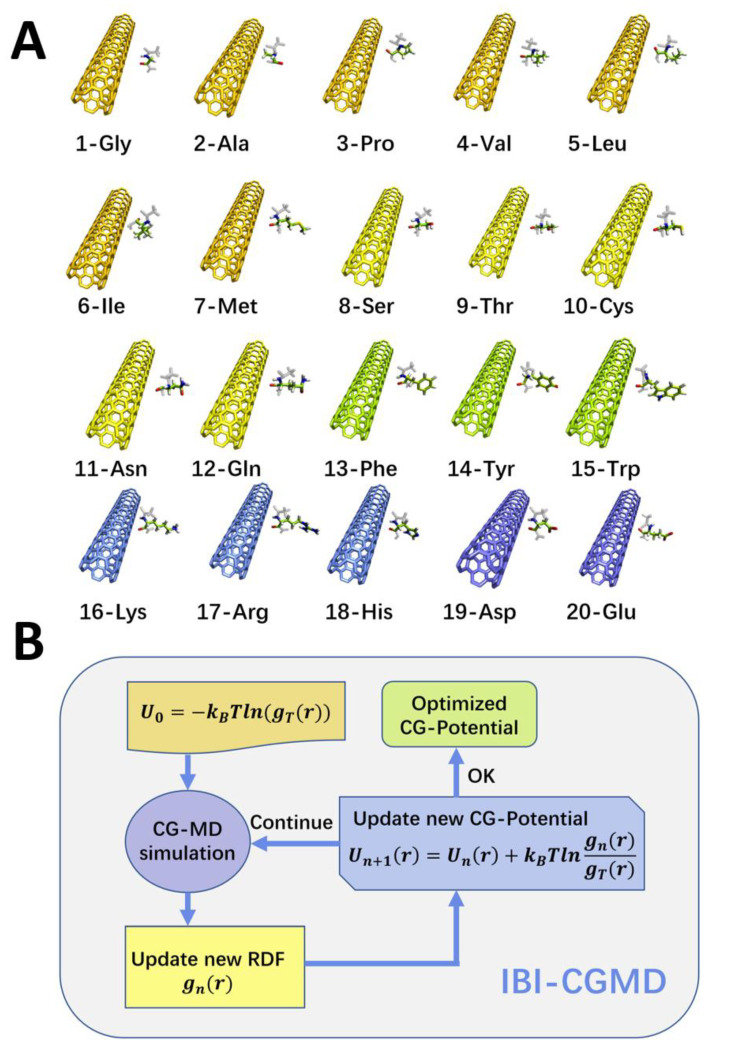
(**A**) Schematic diagram of the 20 amino acids–CNT55L3 simulation system. Amino acids are grouped as: (1) non-polar amino acid group (orange); (2) polar uncharged amino acid group (yellow); (3) aromatic amino acid group (green); (4) positively charged amino acid group (blue); and (5) negatively charged amino acid group (blue–purple part). (**B**) Simple schematic diagram of IBI process, IBI reverse Boltzmann iteration process to extract coarse-grained force field from all-atom trajectories, and $U_{0}$ is obtained from the AAMD.

Our research has constructed 20 molecular systems, namely periodic infinite length CNT55L3 × 20 amino acids. We studied the interaction between carbon nanotube CNT55L3 and 20 amino acids in an aqueous solution (Figure 1A), and each research system was used for three repeated simulation studies: a total of more than 60 simulation research trajectories, each trajectory 200 ns. We hope to conduct objective statistical research from the atomic level to lay the foundation for the next force field extraction. Our research, based on previous studies of molecular dynamics between carbon-based nanoparticles and proteins by other researchers, has grouped 20 amino acids. As shown in Figure 1A, they include a (1) non-polar amino acid group; (2) polar uncharged amino acid group; (3) aromatic amino acid group; (4) positively charged amino acid group; and (5) negatively charged amino acid group. Such a combination arrangement allows us to conduct research more systematically.

The simulation system of CNT and amino acids (CNT55L3 × 20 amino acids) is in an aqueous solution. The simulations were performed using the GROMACS (version 4.6.5) with the Amber03 [37] force field. The SPC [38] water model was applied for water molecules. We use Gromacs software throughout our molecular dynamics research. Our research used the leapfrog algorithm, and the simulation step is 2 fs. In order to ensure that there is sufficient adsorption time between the amino acid and CNT55L3, the duration of each simulation is 200 ns. In the initial stage, the distance between the amino acid and CNT55L3 is about 1.5 nm. Our simulation systems are all NVT systems. The simulated temperature of the system is 330 K, and we use the Berendsen thermostat. The Box parameters of the simulation system are: $L_{x}=4.16\;\mathrm{nm}$, $L_{y}=4.16\;\mathrm{nm}$, $L_{y}=2.93\;\mathrm{nm}$. We use periodic boundary conditions to make our carbon nanotubes infinitely periodic. The long-range Coulomb interactions were treated with the PME [39] method. The van de Waals (vdW) interactions were handled with a smooth cutoff with a distance value of 1 nm. It should be noted that in the CGMD part, most of our parameters are consistent with AAMD. It still used the leapfrog algorithm. The simulation step is the same during the test. When our CGMD system is stable, the longest integration step size can reach 1000 fs, and each iteration must reach more than 100,000 steps. During the CGMD iteration, the temperature was maintained at 330 K using a V-scale thermostat. Semi-empirically, the V-scale thermostat is more suitable than the Berendsen thermostat in CGMD.

The focus of this research is to extract effective coarse-grained force field parameters from the all-atom simulation system of CNT55L3 × 20 amino acids. In order to obtain sufficient interaction conformation sampling in the AAMD part, we mainly made the following three efforts: (a) **The sufficiently large sampling space.** For the CNT part, we use a periodically wireless long tube. Within a certain range of computing resources, the simulation box is L_x = 4.16 nm, L_y = 4.16 nm, L_y = 2.93 nm, which can provide a sufficient sampling space; (b) **The sufficiently long simulation time.** We set the simulation duration at 200 ns to ensure sufficient contact between CNTs and amino acid molecules. The duration of other nanoparticle studies is generally in the tens of nanoseconds. In the previous simulation experiments, we tested the duration ranging from 10 to 50 ns and finally found that the simulation duration of about 200 ns can ensure that the interaction between all amino acids and CNTs can be fully sampled; (c) **The sufficiently high sampling temperature.** The study took a sampling temperature slightly above room temperature. We have tested 270 K, 290 K, 300 K, 330 K, 350 K, etc., of which 330 K is a more suitable sampling temperature; in addition, we have to mention that we have plans to conduct multi-temperature field coupling in the future.

### 2.2. Coarse-Grained Mapping Method

**Figure 2:**
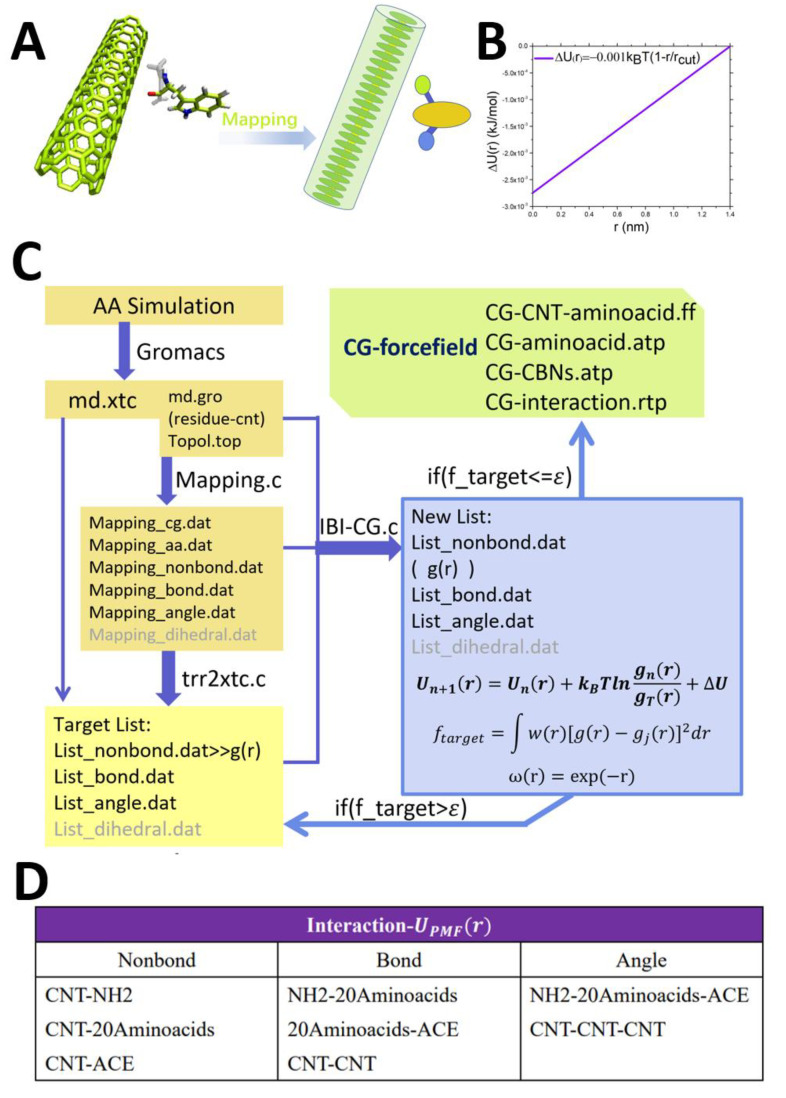
(**A**) Schematic diagram of the coarse-grained method of the simulation system Mapping: (1) CNT part: 10 AAbeads are coarse-grained into one CGbead, 24 CNT-CGbeads in total; (2) Amino acids Part: An amino acid molecule is coarse-grained into one CGbead. Mapping the main chain and side chain of the amino acid to one CGbead. (**B**) Linear addition function. (**C**) Detailed flow chart of IBI-20Aminoacids-CNT55L3, including the interaction process of each file. (**D**) Table: list of the average field potential energy of each initial interaction relationship.

There are two classic coarse-grained methods: (1) center of mass (COM) based on the coarse-grained research object; and (2) based on the structure center of the coarse-grained research. This method is suitable for the geometric structure center and the center of mass of the research object to coincide exactly. In addition, in recent years, the use of clustering to coarse-grain some fluid molecular clusters is also a new method. Our research adopts the method of coarse-grained extraction of the COM coordinates; as shown in Figure 2A, the calculation method of the COM coordinates is:

$$R_{I}=\overset{n}{\underset{i}{\sum }}\frac{r_{i}m_{i}}{\sum_{i}^{n}m_{i}}$$

Among them, $R_{I}$ refers to the position coordinate of each coarse-grained particle (CG-Bead), and $r_{i}$ is the coordinate of the all-atomic particle (AA-Bead) included in each coarse-grained particle. The mass of coarse-grained particles is $M_{I}=\overset{n}{\underset{i}{\sum }}m_{i}$. Our coarse-grained model is divided into two parts: (1) CNT part: 10 AAbeads are coarse-grained into one CGbead, 24 CNT-Cgbeads in total, and periodic boundary conditions are used in the simulation; (2) Amino acids part: An amino acid molecule is coarse-grained into one CGbead. That is to say, we simplify the main chain and side chain of the amino acid to one CGbead. It should be noted here that we have tried to coarse-grain an amino acid molecule into multiple CGbeads, but the result is not good, and the obtained RDF is not smooth enough, which is not conducive to the later extraction of the force field. In addition, the modification groups ACE on both sides coarse-grained separately with NH2 into a CGbead. Our coarse-grained method can be effectively compared with the AAMD process in the CGMD simulation process to extract a more appropriate coarse-grained force field.

### 2.3. Iterative Boltzmann Inversion Method

IBI (Iterative Boltzmann Inversion) is a multi-scale simulation method that has attracted the attention of many researchers in recent years. It can effectively modify the coarse-grained model derived from the mean field [40,41,42,43,44]. IBI is based on the distribution result $g_{Target}\left(r\right)$ obtained by all-atom molecular dynamics simulation, and it extracts the PMF potential energy

$$U_{PMF}=-k_{B}Tln\left(g_{Target}\left(r\right)\right),$$

Then, we use the initial potential energy extracted by the mean field, using the iterative equation

$$U_{n+1}\left(r\right)=U_{n}\left(r\right)+k_{B}Tln\frac{g_{n}\left(r\right)}{g_{T}\left(r\right)}$$

Perform simulation iterations [45,46,47].

The system parameters required by the system we studied are: non-bond interaction, bond interaction, and angle interaction [19,41,42,47,48,49]:

$$U_{Total}\left(r\right)=U_{Nonbond}\left(r\right)+U_{Bond}\left(l\right)+U_{Angle}\left(\theta \right)$$

$$P\left(r,l,\theta \right)=P_{Nonbond}\left(r\right)P_{Bond}\left(l\right)P_{Angle}\left(\theta \right)$$

According to R. L. Henderson’s uniqueness theorem [50], we can conclude that the particle distribution of the system is positively correlated with its potential energy function. Henderson’s theorem is the theoretical premise of the potential energy function-like coarse-grained method based on the radial distribution function. The various interactions (Figure 2D Table) are as follows:

$$P_{Nonbond}\left(r\right)=g_{Target}\left(r\right)\propto \exp\left(-\frac{U_{Nonbond}\left(r\right)}{k_{B}T}\right)$$

$$P_{Bond}\left(l\right)\propto \exp\left(-\frac{U_{Bond}\left(l\right)}{k_{B}T}\right)$$

$$P_{Angle}\left(\theta \right)\propto \exp\left(-\frac{U_{Angle}\left(\theta \right)}{k_{B}T}\right)$$

$U_{Bond}\left(l\right)$ and $U_{Angle}\left(\theta \right)$ are the non-iterative part of the potential energy extracted by PMF, and the $U_{Nonbond}\left(r\right)$ also needs subsequent IBI optimization:

$$U_{Nonbond}\left(r\right)=-k_{B}Tln\left(g_{Target}\left(r\right)\right)$$

$$U_{Bond}\left(l\right)=-k_{B}Tln\left(P_{Bond}\left(l\right)\right)$$

$$U_{Angle}\left(\theta \right)=-k_{B}Tln\left(P_{Angle}\left(\theta \right)\right)$$

Among them, $U_{Nonbond}\left(r\right)$ can be used as the initial potential energy for subsequent IBI iterations, namely

$$U_{Nonbond}^{0}\left(r\right)=U_{Nonbond}\left(r\right)=-k_{B}Tln\left(g_{Target}\left(r\right)\right).$$

As shown in Figure 1B, the IBI iteration (n_Iterative) method of non-bond interaction is as follows:

$$U_{Nonbond}^{n+1}\left(r\right)=U_{Nonbond}^{n}\left(r\right)+k_{B}Tln\left(\frac{g^{n}\left(r\right)}{g_{Target}\left(r\right)}\right)$$

where *n* is the number of IBI iterations, and $g_{Target}\left(r\right)$ is the target radial distribution function of the system. In the iterative process of IBI, part of the force calculation method [51] is:

$$F_{Nonbond}\left(r\right)=-\frac{1}{\beta }\frac{d}{dr}\ln(g^{n}\left(r\right))$$

$$F_{Bond}\left(r\right)=-\frac{1}{\beta }\frac{d}{dl}\ln\left(P_{Bond}\left(l\right)\right)$$

$$F_{Angle}\left(\theta \right)=-\frac{1}{\beta }\frac{d}{d\theta }\ln\left(P_{Angle}\left(\theta \right)\right)$$

Among them, β=1kBT.

The general process of the classic IBI is as shown in Figure 1B. First, the distribution and interaction function between coarse-grained atoms are extracted by the all-atom simulation, and then, the coarse-grained molecular dynamics simulation (CGMD) is performed for a simulation: that is, an iteration. Then, we extract the corresponding new distribution. If the new distribution fits the original target distribution function, the iteration ends; if the error between the new distribution and the original target distribution function does not fit the corresponding requirements, we add the function obtained from the iteration to the next iteration of the potential energy function. The next iteration will be performed until the fitting accuracy meets the research requirements.

In the specific IBI simulation iteration process, there will be many detailed problems that need to be resolved: for example, how to make the formats of the system files under study recognize each other, and how to keep the simulation unit during the transition from AA to CG as the appropriate choice. As shown in Figure 2C, the details of IBI’s key documents are shown in the flowchart. Among them, our AA part of the simulation used Gromacs molecular dynamics simulation software. The coarse-grained model uses md.gro and topol.top in Gromacs (topological files related to molecular structure characteristics), from which we can read the mass, atomic number, charge, and so on of each AA atom. In the process of extracting the AA target distribution function, we used the official supporting analysis tool XdrFile1.1 of the Gromacs software, in which trr2xtc.c is the trajectory analysis program that comes with the software package. In addition, in the process of IBI-CGMD, we programmed a set of iterative procedures ourselves. Each group of amino acids has undergone more than 100 iterations. The integration step length of each iteration is 0.001 ps, and each iteration runs at least 100,000 steps so that we can obtain more appropriate statistics. The convergence decision function in the iterative process is:

$$f_{Target}=\int w\left(r\right){\left[g\left(r\right)-g_{Target}\left(r\right)\right]}^{2}dr$$

Among them, $w\left(r\right)=\exp\left(-r\right)=e^{-r}$ is the system weight function [26]. In addition, in order to make the system potential energy update more smoothly, we use interpolation to smooth the potential energy curve. Then, we use a semi-empirical linear function to superimpose the potential energy update process, as shown in Figure 2B:

$$U_{Nonbond}^{n+1}\left(r\right)=U_{Nonbond}^{n}\left(r\right)+k_{B}Tln\left(\frac{g^{n}\left(r\right)}{g_{Target}\left(r\right)}\right)+\Delta U\left(r\right)$$

$$\Delta U\left(r\right)=\alpha \left(1-\frac{r}{r_{cut}}\right)$$

$$\alpha =-0.001*k_{B}T$$

$$r_{cut}=Value\left(r\_cutoff\right)$$

Among them, $\Delta U\left(r\right)=\alpha \left(1-\frac{r}{r_{cut}}\right)$ is a linear function [44]; when *r* = $r_{cut}$, the value of the function is 0; when *r* = α, the value of the function is α, that is α = −0.001 ∗ $k_{B}T$. Among them, the terms of *g*(*r*) and $\frac{r}{r_{cut}}$ are non-dimensional terms, so the units of the above three formulas are the same as $k_{B}T$. We used the GSL (GNU Scientific Library) function package in our research. Furthermore, it should be noted that the molecular dynamics units of IBI-CGND are the same as those in Gromacs. Above, we have introduced in detail the specific simulation iteration process of IBI and the details of various parameters.

## 3. Results and Discussion

### 3.1. Initial PMF of CNT55L3-20aminoacids

Next, we will introduce the results of the average field potential energy extraction of the system from the aspects of Non-Bond, Bond, and Angle, where U-PMF is the average field potential energy derived from the target distribution.

**Figure 3:**
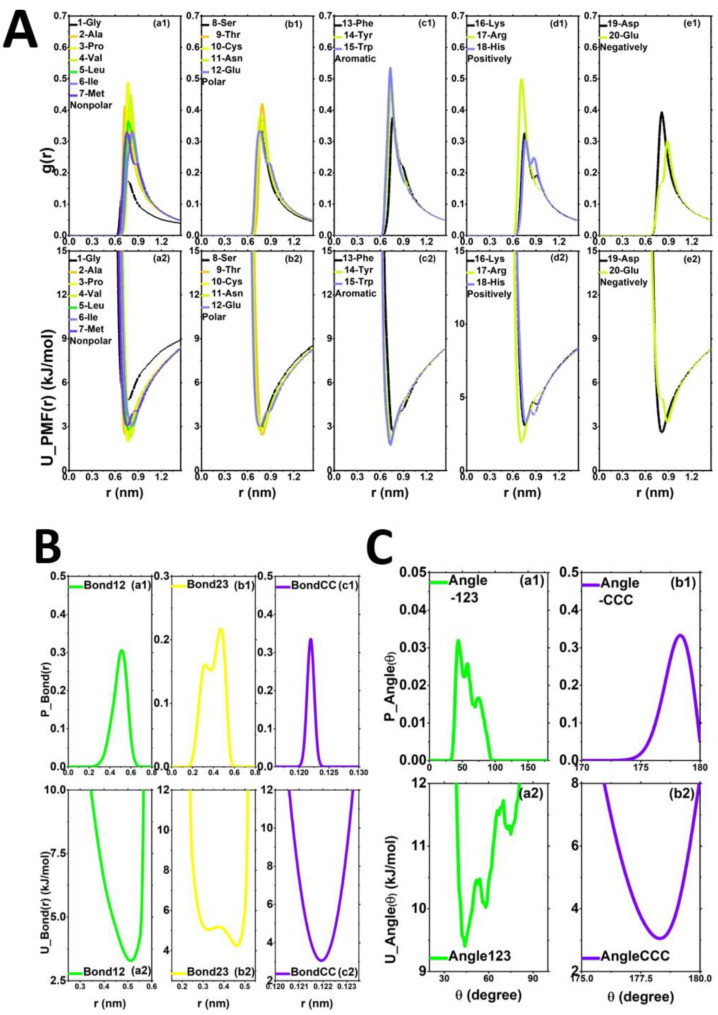
(**A**) The radial distribution functions between different types of amino acids and carbon nanotubes and their potential energy functions extracted by full-atom simulation: non-polar amino acid group RDF (**a1**) and its U_PMF(r) (**a2**); the RDF (**b1**) of the polar uncharged amino acid group and its U_PMF(r) (**b2**); aromatic amino acid group RDF (**c1**) and its U_PMF(r) (**c2**); the positively charged amino acid group RDF (**d1**) and its U_PMF(r) (**d2**); the negatively charged amino acid group RDF (**e1**) and its U_PMF(r) (**e2**). (**B**) The bond distribution functions extracted by the all-atom simulation and its potential energy function (take CNT55L3-15-Trp as an example): NH2-amino acids, (**a1**) P_Bond(r), (**a2**) U_Bond(r); amino acids-ACE, (**b1**) P_Bond(r), (**b2**) U_Bond(r); CNT-CNT, (**c1**) P_Bond(r), (**c2**) U_Bond(r). (**C**) The distribution of the angle distribution function extracted by the all-atom simulation and its potential energy function (take CNT55L3-15-Trp as an example): NH2-amino acids-ACE, (**a1**) P_Angle(θ), (**a2**) U_Angle(θ); CNT-CNT-CNT, (**b1**) P_Angle(θ), (**b2**) U_Angle(θ).

(1) **The initial distribution and PMF of the non-bond group.** Figure 3A is a schematic diagram of the non-bond radial distribution function *g*(*r*) between 20 amino acids and CNT55L3 and the potential energy function of each group. We use the coarse-grained distribution of centroids to accurately reflect the adsorption characteristics of each amino acid and CNT55L3. Among them, the RDF of aromatic amino acids (Figure 3A(c1,c2)) has a higher first peak. There are also differences between the different amino acids within. In Figure 3A(d1,d2), we can observe that the potential energy depth of 17-Arg is comparable to that of aromatic amino acids. This is very necessary, because this part of the potential energy is the same as the initial potential energy curve for the next iteration of IBI.
(2) **The initial distribution and PMF of the bond group.**Figure 3B describes the distribution of the bond group extracted by the AA system and the potential energy curve. Among them, Figure 3B(a1,a2) is the bond distribution function and potential energy curve of NH2-Trp; Figure 3B(b1,b2) is the bond distribution function and potential energy curve of Trp-ACE; Figure 3B(c1,c2) shows the bond distribution function and potential energy curve of CNT-CNT. The extraction of this part of the potential energy curve is the force field parameter that needs to be used in the next iteration of IBI.
(3) **The initial distribution and PMF of the angle group.** Figure 3C describes the distribution function of the angle part and the potential energy curve extracted by the AA system. Among them, Figure 3C(a1,a2) is the distribution of the angle part of NH2-Trp-ACE and the potential energy curve; Figure 3C(b1,b2) is the distribution of the angle part of CNT-CNT-CNT and the potential energy curve. Generally speaking, this part of the average field potential can either choose to iterate or not to iterate. Since the focus of our research is on the non-bonded interaction energy of CNT-amino acids, we will not do iterative optimization for this part of the content.

### 3.2. The Iteration Process of CNT55L3-20aminoacids

A successful IBI iterative process is based on whether the radial distribution functions generated by AAMD and CGMD in the iterative process can overlap well. The higher the accuracy of the fitting function $f_{Target}$, the closer the dynamics of our IBI iterative system and AAMD. As shown in Figure 7-Table(C), the accuracy of the fitting function $f_{Target}$ can reach $e^{-4}$–$e^{-5}$ (that is $10^{-4}$–$10^{-5}$, and we use the double format). Our iterative fitting results can be considered to have reached a higher accuracy compared with previous IBI-related work.

#### 3.2.1. IBI-Representative Amino Acid Research

**Figure 4:**
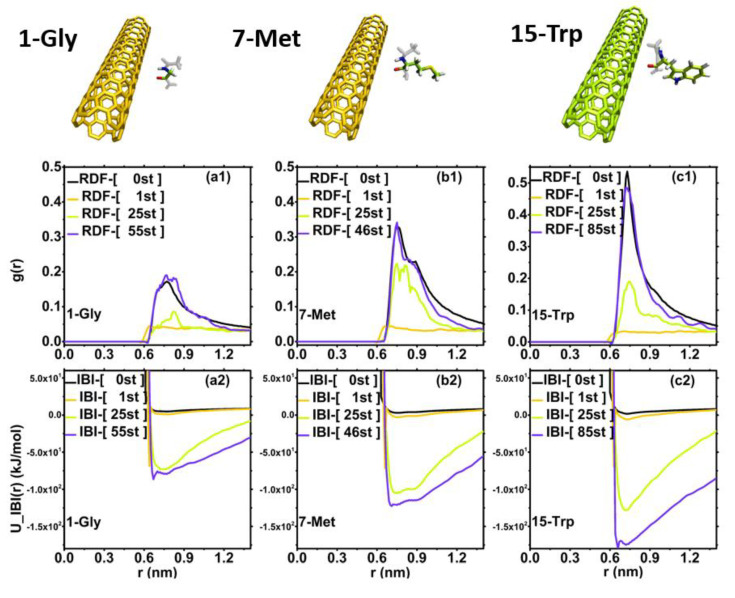
Representative amino acid studies: (1) short-chain non-polar amino acid 1-Gly, (**a1**) RDF iteration, (**a2**) IBI potential energy iteration; (2) long-chain non-polar amino acid 7-Met, (**b1**) RDF iteration, (**b2**) IBI potential energy iteration; (3) Aromatic amino acid 15-Trp, (**c1**) RDF iteration, (**c2**) IBI potential energy iteration.

We selected three representative amino acids to show the difference in CNT adsorption strength. These three amino acids are (1) short-chain non-polar amino acid 1-Gly, (2) long-chain non-polar amino acid 7-Met, and (3) 15-Trp, an aromatic amino acid. As shown in Figure 4, (a1–c1) represent their respective RDF iteration processes, and (a2–c2) represent their respective IBI potential energy iteration processes. Among them, the black line is IBI-[0st], the orange line is IBI-[1st], the green line is IBI-[25st], and the blue–purple line is the number of iterations each reached a certain fitting accuracy. The iteration accuracy of the three amino acids is: (1) the number of optimization iterations for 1-Gly is IBI-[55st], and the fitting accuracy is f_target = 1.405139e-04; (2) the number of optimization iterations for 7-Met is IBI-[46st], and the fitting accuracy is f_target= 2.522036e-04; (3) the number of optimization iterations of 15-Trp is IBI-[85st], and the fitting accuracy is f_target= 2.881077e-04. We can observe that as the number of iterations increases, the RDF of amino acids is becoming closer and closer to $g_{Target}\left(r\right)$, and at the same time, its U_IBI(r) iteration potential becomes deeper.

In addition, we can also clearly observe the following from Figure 4: (1) from the perspective of RDF first-peak: RDF-1-Gly < RDF-7-Met < RDF-15-Trp; (2) from the perspective of the potential well depth of the interaction between the three amino acids and CNT, the aromatic amino acid 15-Trp is significantly deeper than other amino acids, while the long-chain non-polar amino acid 7-Met is deeper than the short-chain non-polar amino acid 1-Gly. This also shows that during the interaction between amino acids and carbon nanotubes, the π–π interaction has a greater influence on the adsorption strength than the hydrophobic interaction. This conclusion coincides with the results of AAMD’s kinetic analysis, which also shows that our multi-scale IBI research results are trustworthy.

#### 3.2.2. The Distribution during IBI Iteration

**Figure 5:**
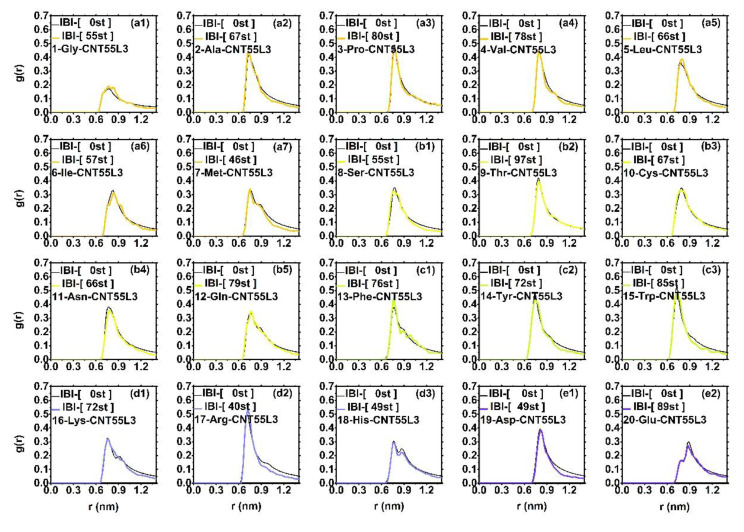
The radial distribution function and target radial distribution function between 20 amino acids and CNT55L3 obtained after IBI iterative optimization. The black lines in each figure are their respective target RDF, and (**a1**–**a7**) are the non-polar RDF of the amino acid group, the line color is orange; (**b1**–**b5**) is the RDF of the polar uncharged amino acid group, the line color is yellow; (**c1**–**c3**) is the RDF of the aromatic amino acid group, the line color is green; (**d1**–**d3**) is the RDF of the positively charged amino acid group, the line color is blue; (**e1**,**e2**) is the RDF of the negatively charged amino acid group, the line color is purple.

Figure 5 shows the iterative process of the radial distribution function of the IBI system between 20 amino acids and CNT55L3. The part of the black line in each data graph is the target distribution function $g_{Target}\left(r\right)$ between each amino acid and CNT55L3. Among them, in order to facilitate the reader to observe the data, we set the amino acid color of the same group to be consistent. We can clearly observe that the RDFs between these 20 amino acids and CNTs are all well fitted to the target distribution function $g_{Target}\left(r\right)$ within the allowable precision of the error. When slightly flawed, such as 01-Gly-CNT55L3 compared with its target distribution function $g_{Target}\left(r\right)$, the degree of coincidence is not so good, but its target distribution function $g_{Target}\left(r\right)$ still reaches 1.405139e-04, which is an acceptable error scope. This fully shows that our IBI iterative process is effective. Here, we have completed the fitting of the target distribution function between 20 amino acids and CNT55L3 carbon nanotubes.

#### 3.2.3. Potential Energy in the Iterative Process of IBI

**Figure 6:**
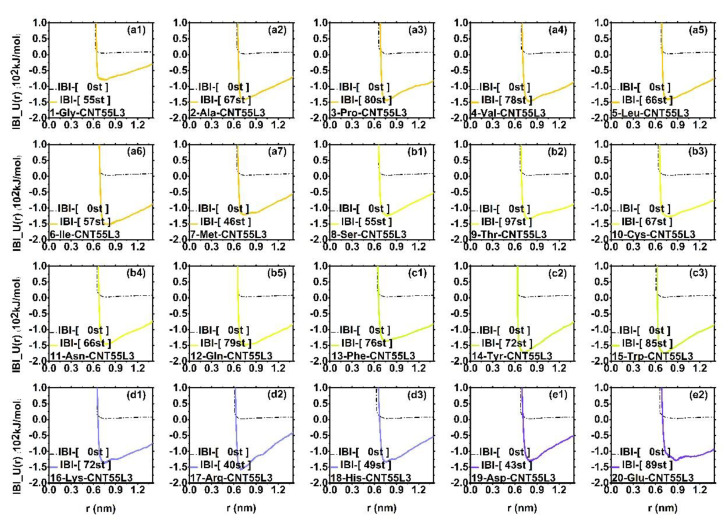
The non-bond potential energy function and initial non-bond potential energy function (PMF) between 20 amino acids and CNT55L3 obtained after IBI iterative optimization. The black dotted lines in each figure are their respective initial non-bond PMF, and (**a1**–**a7**) are the non-polar PMF of the amino acid group, the line color is orange; (**b1**–**b5**) is the non-bonding PMF of the polar uncharged amino acid group, the line color is yellow; (**c1**–**c3**) is the non-bond PMF of the aromatic amino acid group, the line color is green; (**d1**–**d3**) is the non-bonding PMF of the positively charged amino acid group, and the line color is blue; (**e1**,**e2**) is the non-bond PMF of the negatively charged amino acid group, and the line color is purple.

**Figure 7:**
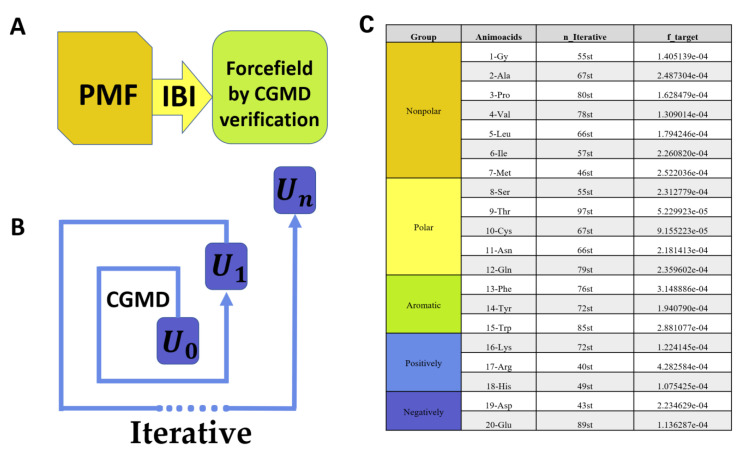
(**A**) Initialized by PMF, it then obtains a proven force field with IBI; (**B**) IBI through CGMD to generate a new potential energy function every iteration; (**C**) The accuracy of the decision function between the initial target radial distribution function of amino acids and CNT55L3 after IBI iterative optimization and the radial distribution function obtained by the best iteration.

Figure 6 shows the change of the non-bond potential energy function of the 20 amino acids and CNT55L3 after the iteration of the radial distribution function of the IBI system within the acceptable accuracy range. We can observe that the potential energy of the 20 amino acids has become deeper. That is to say, the potential energy function of the interaction between the 20 amino acids and the CNT55L3 has been well optimized so that the coarse-grained kinetics process can be simulated in all atoms; as shown in the table in Figure 7C, there is a similar effective distribution within the accuracy range.

The above is the data recording and display of the entire IBI iteration process of 20 amino acids and CNT55L3. As shown in the table in Figure 7C, we see that the simulation accuracy can reach e-4 to e-5, which is a relatively high fitting accuracy in related studies using IBI. The results of our research work also show that it is very appropriate to choose IBI as the research method for the force field extraction of carbon nanotubes and 20 standard amino acids, and the coarse-grained force field parameters within a certain accuracy error range are obtained. In addition, it should be noted that PMF derived from AAMD is not tested by CGMD. As shown in Figure 7A,B, after being initialized by PMF, it then obtains a proven force field with IBI. The interaction relationship after IBI iterative optimization is the effective potential energy that can adapt to CGMD, which is consistent with the results obtained by AAMD.

## 4. Summary and Outlook

Our work investigated the coarse-grained interaction between 20 amino acids and the representative carbon nanotube CNT55L3. Our research process is based on the distribution result $g_{Target}\left(r\right)$ obtained by all-atom molecular dynamics simulation: first, it extracts the PMF potential energy, and then, it uses the initial potential energy extracted by the average field to perform simulation iterations using IBI. Our research results have been carried out for more than 100 iterations, and finally, the RDF obtained by the coarse-grained simulation (CGMD) can effectively coincide with the result of the all-atomic molecular dynamics simulation (AAMD). In addition, our work lays the foundation for force field research for the simulation of coarse-grained super-large proteins and other important nanoparticles.

However, our current research work still has certain limitations: **(1) The particularity of the research system.** Various special parameters in the IBI program we studied were designed for this particular system. This system is different from the traditional IBI multi-scale approach. Traditional IBI research systems are normally fluid systems and polymer systems. Multi-scale studies of fluid systems are mostly point-to-point CG-Bead. Multi-scale studies of polymer systems generally use a one polymer-unit as a CG-Bead, and polymer chains are mostly flexible. Our research system is special, which is a rigid CNT and a flexible modified amino acid short peptide; **(2) The specificity of the operating parameters.** Based on the particularity of our research, the operating parameters of our research are also adjusted around the simulation system. For example, in our study, we used a V-scale thermostat instead of the Berendsen thermostat, which is more commonly used in current MD studies; **(3) The limitations of the IBI program.** Currently, our IBI program is not comprehensive enough to provide multiple kinetic options. For example, the multi-scale software VOTCA can provide multiple kinetic methods including MD and MC. Our research focuses on IBI, while other software, such as VOTCA, Cafemol, etc., can provide IBI, FM and other multi-scale methods. Therefore, for the learning of multi-scale methods, it is a worthwhile choice for researchers to use the other mature software.

Looking forward to the future, there are three challenges. (I) First, we need to learn how to normalize the coarse-grained force field parameters between the 20 standard amino acids and carbon nanotubes that we have obtained and combine them with some mainstream molecular dynamics simulation software. It is convenient for molecular dynamics simulation researchers to use our force field parameters and then provide a new way to study the interaction between super-large proteins and carbon-based nanoparticles. (II) Second, it is important to learn how to extend this set of IBI force field extraction between carbon-based nanoparticles and amino acids to other nanoparticles, such as gold nanoparticles, silicon-based nanoparticles, etc. (III) Third, we also hope that the IBI program can be combined with machine learning and deep learning to optimize the current IBI algorithm in the future.

## Sample Availability

Samples of the compounds are available from the authors.
